# A novel prognostic mRNA/miRNA signature for esophageal cancer and its immune landscape in cancer progression

**DOI:** 10.1002/1878-0261.12902

**Published:** 2021-02-08

**Authors:** Yue Zhao, Li Xu, Xinyu Wang, Shuai Niu, Hezhong Chen, ChunGuang Li

**Affiliations:** ^1^ Department of Thoracic Surgery Changhai Hospital Second Military Medical University Shanghai China; ^2^ Department of Thoracic Surgery Shanghai Pulmonary Hospital School of Medical Tongji University Shanghai China; ^3^ Department of Vascular Surgery Peking Union Medical College Hospital Chinese Academy of Medical Sciences and Peking Union Medical College Beijing China

**Keywords:** drug sensitivity, immune subtype, miRNAs, prognosis, tumor microenvironment, tumor mutation burden

## Abstract

Mounting evidence shows that MicroRNAs (miRNAs) and their target genes are aberrantly expressed in many cancers and are linked to tumor occurrence and progression, especially in esophageal cancer (EC). This study purposed to explore new biomarkers related to the prognosis of EC and to uncover their potential mechanisms in promoting tumor progression. We identified 162 differentially expressed miRNAs and 4555 differentially expressed mRNAs in EC. Then, a risk model involving three miRNAs (miR‐4521, miR‐3682‐3p, and miR‐1269a) was designed to predict prognosis in EC patients. Furthermore, 7 target genes (Rho GTPase‐activating protein 24, Chromobox 3, Contactin‐associated protein 2, ELOVL fatty acid elongase 5, LIF receptor subunit alpha, transmembrane protein 44, and transmembrane protein 67) were selected for Gene Ontology and Kyoto Encyclopedia of Genes and Genomes analyses to reveal their potential mechanisms in promoting EC progression. After a series of correlation analyses, miRNA target genes were found to be significantly positively or negatively associated with immune infiltration, tumor microenvironment, cancer stemness properties, and tumor mutation burden at different degrees in EC. To further elucidate the role of miRNA signature in cancer progression, we performed a pan‐cancer analysis to determine whether these genes exert similar effects on other tumors. Interestingly, the miRNA target genes altered expression on tumor immunity; however, pan‐cancer progression was the same as that of EC. Thus, we explored the immune landscape of the miRNA signature and its target genes in EC and pan‐cancer. These findings demonstrated the versatility and effectiveness of our model in various cancers and provided a new direction for cancer management.

## Graphical abstract

MicroRNAs and their target genes play pivotal role in esophageal cancer (EC) progression. Here, we established a miRNA‐based prediction model in predicting patient prognosis and explored their target genes. We further detected the role of miRNA target genes in immune landscape in EC and pan‐cancer, which could provide with a new direction for cancer management in EC.

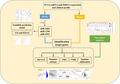

**Abbreviations**
ARHGAP24: Rho GTPase‐activating protein 24
CBX3: Chromobox 3
CNTNAP2: Contactin‐associated protein 2
ELOVL5: ELOVL fatty acid elongase 5
LIFR: LIF receptor subunit alpha
TMEM44: transmembrane protein 44
TMEM67: transmembrane protein 67

## 1 Introduction

Esophageal cancer (EC) is the 8th most common cancer and the leading cause of cancer‐associated deaths globally [1]. Despite great advances in the diagnosis and treatment of EC, effective biomarkers that facilitate precision diagnosis and therapy are lacking [2]. MicroRNAs (miRNAs), small endogenous noncoding RNAs, can regulate gene expression in the posttranslational level by directly binding to target mRNAs [3]. MiRNAs have been implicated in a variety of biological processes (BP), thus promote tumor progression, including EC [4]. For example, in our previous study, we found that miR‐34a‐5p could directly target LEF1 and promote epithelial–mesenchymal transition and progression in esophageal squamous cell carcinoma [5]. Some studies also confirmed that miRNA profiles could be used in predicting the survival outcome of EC patients [6]. The above findings indicate that miRNAs may serve as a promising biomarker for diagnosis, prediction of survival, and precision treatment in cancers.

Although the crucial role of miRNAs as biomarkers in various cancers has been extensively elucidated, the potential mechanisms of miRNAs and their target genes in tumor progression and poor patient prognosis remain elusive. Growing evidence has shown that tumor microenvironment (TME) participates in tumor progression and metastasis of various cancers [7]. All components of TME including cancer cells and noncancer cells (infiltration immune cells, stromal cells, and extracellular matrix) have been revealed to play important roles in crosstalk with tumor cells to affect tumor progression and invasion. Numerous studies have shown that tumor‐associated stromal cells are involved in the progression and invasion of various cancers [8]. The response of immune treatment could be evaluated by nonimmune cell components in TME. For instance, the elimination of immune cells in TME and the resistance to chemotherapy is affected by transforming growth factor‐β (TGF‐β) secreted by fibroblasts [9].

Also, immune cells infiltrated in TME can kill tumor cells via various mechanisms. More importantly, miRNAs also pose their indispensable role in the regulation of TME. Recent studies have demonstrated that miRNAs and their target genes are expressed in both stromal and tumor cells, promoting tumor development and progression. Thus, both miRNAs and target genes could serve as modulators between different types of infiltrated immune and tumor cells. However, miR‐155 which is a widely explored miRNA in immune cells was shown to be down‐regulated in tumor‐associated macrophages (TAMs) which consequently promoted the expression of IL‐10 by targeting C/EBPβ, hence enhanced its immunosuppressive function in cancer [10].

Cancer stem cells (CSCs), generated during cancer progression, are considered for cancer recurrence, chemotherapy resistance, and tumor progression [11]. Our previous study explicitly elucidated the role of transcriptional factor LEF1 and protein arginine methyltransferase‐1 in the regulation of cancer stem‐like properties and resistance to chemotherapy in esophageal squamous carcinoma cell [12, 13]. Moreover, CSCs were reported to have tight crosstalk with TME and immune response. Evidence from ovarian cancer showed that marrow‐derived suppressor cells, an inhibitor of other immune cells in TME, could promote phenotype of CSCs by inducing miR‐101 expression [14]. CSCs from patients with head and neck squamous cell carcinoma and melanoma were confirmed to recruit regulatory T cells with immunosuppressive and tumor‐promoting effects and promoted *in vitro* proliferation [15]. Moreover, miRNAs could correlate with cancer stem‐like properties in various tumors. Specifically, miR‐181b directly binds to STAT3 and activates downstream CYLD pathways regulating the proliferation of CSCs in esophageal squamous cell carcinoma [16]. Therefore, exploring the role of miRNAs and target genes in CSCs would facilitate an in‐depth understanding of tumor progression and precision therapy.

Immune therapy is recently considered as a promising novel method for EC treatment in terms of immune checkpoint inhibitors, tumor vaccine, and adoptive T‐cell treatment. Notably, programmed cell death protein‐1 (PD‐1) and programmed cell death protein ligand 1 (PD‐L1) blockages were the widely used immune checkpoint inhibitors. Tumor mutation burden (TMB), which was revealed to be related to the generation of neoantigens, has been applied to predict the response to PD‐1 and PD‐L1 blockages in various tumors. Recent studies also demonstrated that differentially expressed miRNA‐based signature and its target genes were associated with TMB levels in lung adenocarcinoma [17]. Thus, exploring a more effective miRNA signature is imperative to facilitate the prediction of immune therapy in EC.

In the present study, we constructed and validated a three miRNA‐based signature that effectively predicted the overall survival (OS) of EC patients in the TCGA database. Functional of Gene Ontology (GO) and Kyoto Encyclopedia of Genes and Genomes (KEGG) analysis indicated some potential biological functions and signaling pathways involved in the three miRNA signature. We further explored the immunological role of the three miRNAs and target genes in EC patients. Moreover, we detected whether three miRNA markers and their target genes in pan‐cancer had the same prognostic and immunological effects in EC, which could facilitate an in‐depth understanding of tumor progression and precision immune treatment.

## 2 Materials and methods

### 2.1 Data download and processing

We downloaded RNA‐seq data of mRNA and miRNA expression profile, clinical information, and single nucleotide polymorphism data of EC from the TCGA database (https://cancergenome.nih.gov/). The mRNA data of 171 samples included 160 tumor samples and 11 normal samples. The miRNA data of 198 samples included 185 tumor samples and 13 normal samples. All mature miRNA sequences were downloaded in Fasta format from the miRBase database (http://www.mirbase.org/) [18] to obtain sequence data. The TCGA pan‐cancer data were downloaded from the UCSC Xena database (https://xenabrowser.net/datapages/) [19], including RNA‐seq, patients' clinical data, stemness scores based on mRNA (RNAss) and DNA methylation (DNAss), and immune subtypes. The TCGA pan‐cancer data included 33 cancer types, and they are ACC, BLCA, BRCA, ESCA, CCA, CESC, COAD, DLBC, GBM, HNSC, KICH, KIRC, KIRP, LAML, LGG, LIHC, RCA, LUAD, LUSC, MESO, OV, PAAD, PCPG, PRAD, READ, SARC, SKCM, TGCT, THCA, THYM, UCEC, UCS, and UVM. A total of 11 058 samples were enrolled in our study, and the number of samples in each cancer type ranged from 45 for CCA to over 1000 for breast cancer. Notably, 18 types of cancers had more than five normal samples and were used to assess altered gene expression profiles.

### 2.2 Identification of DEGs, DEMs, and their association with patient prognosis

The ‘edgeR’ package [20] was used to normalize raw count data and to compare differential expressed genes (DEGs) and differential expressed miRNAs (DEMs) between tumor samples and normal samples, with a false discovery rate (FDR) < 0.05, and |log_2_FC| > 1. Then, we combined standardized and differentially expressed data with clinical information. Subsequently, we used the ‘caret’ package [21] to randomly group all samples with combined information into the training dataset and test dataset with a ratio of 0.7 and further applied the univariate Cox regression analysis to find prognosis related miRNAs in the training group. A total of 14 miRNAs were selected and were all subjected to multivariate Cox regression analysis followed by LASSO analysis. Three miRNA‐based signature was finally filtered, and the risk score was calculated by the expression value of each miRNA and their coefficient. Consequently, the Kaplan–Meier curve was used to analyze the EC patient prognosis based on the median value of risk score in the training, test, and all patient groups. The predictive power of the miRNA signature was evaluated by AUC of 3 years dependent ROC curve using the ‘survivalROC’ package [22]. The relationship of the risk score based on the three miRNA signature and clinical features (gender, age, stage, T stage, lymph nodes invasion, and metastasis) with patient prognosis was analyzed through univariate and multivariate Cox regression.

### 2.3 Predicting target genes of three miRNAs and potential functions

Target genes of three miRNAs were predicted using TargetScan (http://www.targetscan.org/) [23], miRDB (http://www.mirdb.org/miRDB/) [24], and miRWalk (http://mirwalk.umm.uni‐heidelberg.de/) [25] online analysis tools. The candidate genes presented in three databases were regarded as potential miRNA target genes. Meanwhile, cytoscape 3.6.0 software (Institute for Systems Biology, Seattle, WA, USA) [26] was utilized to demonstrate regulation relationships between miRNAs and target genes. To clarify whether the target genes of these miRNAs are likely to participate in the progression of EC, we took the intersections of these target genes and differentially expressed genes, then filtered the intersected genes with the criteria of expressing negatively association with corresponding miRNAs. KEGG signaling pathway and GO enrichment analysis were adopted to reveal potential mechanisms in these intersection genes using ‘clusterProfiler’ package in R [27]. Gene set enrichment analysis (GSEA) was also analyzed using ‘clusterProfiler’ package.

### 2.4 Tumor microenvironment analysis

The ESTIMATE immune, stromal, and estimate scores were used to analyze the infiltration levels of both immune and stromal cells and the purity of tumors in various cancers [28]. This novel algorithm was based on the expression profiles of the TCGA database and proved to be effective in prediction. The association between target gene expression and those scores was tested with Spearman correlation. Moreover, we detected six immune types (C1‐C6) involved in EC and pan‐cancer samples. The Analysis of variance (ANOVA) was used to test the correlation between immune subtypes and target gene expression obtained from TCGA EC and pan‐cancer data. Cancer stem cell‐like properties of each patient obtained from epigenetic and transcriptomic data were used to measure stemness features of tumor cells. The correlation between stemness characteristics and target genes was tested with Spearman analysis.

### 2.5 Evaluating immune infiltration cells in the tumor microenvironment

Recently, Aaron *et al*. developed a new algorithm to analyze 22 types of immune cells involved in the TME and named it CIBERSORT [29]. In our study, we calculated the proportion of 22 tumor‐infiltration immune cells in each EC and pan‐cancer patients based on CIBERSORT score. Moreover, the association between target gene expression and immune infiltration score was assessed to determine which immune cells are significant in different types of cancers.

### 2.6 Drug sensitivity analysis

The NCI‐60 database, containing data from 60 cancer cell lines, was analyzed by cellminer website (https://discover.nci.nih.gov/cellminer/) [30, 31]. The expression status of target genes and *z*‐score for cell sensitivity data (GI50) was downloaded from the website and assessed through Pearson correlation analysis to determine the correlation between target gene expression and drug sensitivity.

### 2.7 Tumor mutation burden analysis

After successfully downloading somatic mutation data from the UCSC database, we calculated the mutation frequency with the number of variants/the length of exons (38 million) for each patient via Perl scripts based on the JAVA9 platform. Then, we used Spearman correlation analysis to evaluate the association between target gene expression and TMB information in each patient of EC and pan‐cancer.

### 2.8 Statistical analysis

All statistical analyses were based on R language 3.6.1 version and the attached packages. Wilcox test was used to identify differentially expressed genes and differentially expressed miRNAs. Comparisons of gene expression in all the tumors across all cancer types and between the normal and tumors in the 18 cancer types which had more than five associated adjacent normal samples were performed using linear mixed‐effects models. Continuous variables were analyzed using Student's *t*‐test. ANOVA was used to test the association between gene expression and immune infiltrate subtypes and cancer subtypes. Log‐rank tests and Kaplan–Meier curve were used to analyze the association between gene or miRNAs expression and patient OS. Spearman correlation method was used to calculate correlation between two variables, such as target genes and all immune‐related traits. Univariate or multivariate Cox regression analyses were performed to evaluate correlation of miRNAs or gene expression with patient survival. The hazard ratio (HR) and 95% confidence interval (CI) were calculated to identify genes associated with OS. *P* < 0.05 was considered statistically significant.

## 3 Results

### 3.1 Identification of differential expressed miRNAs (DEMs) and genes (DEGs) in ESCC

**Fig. 1:**
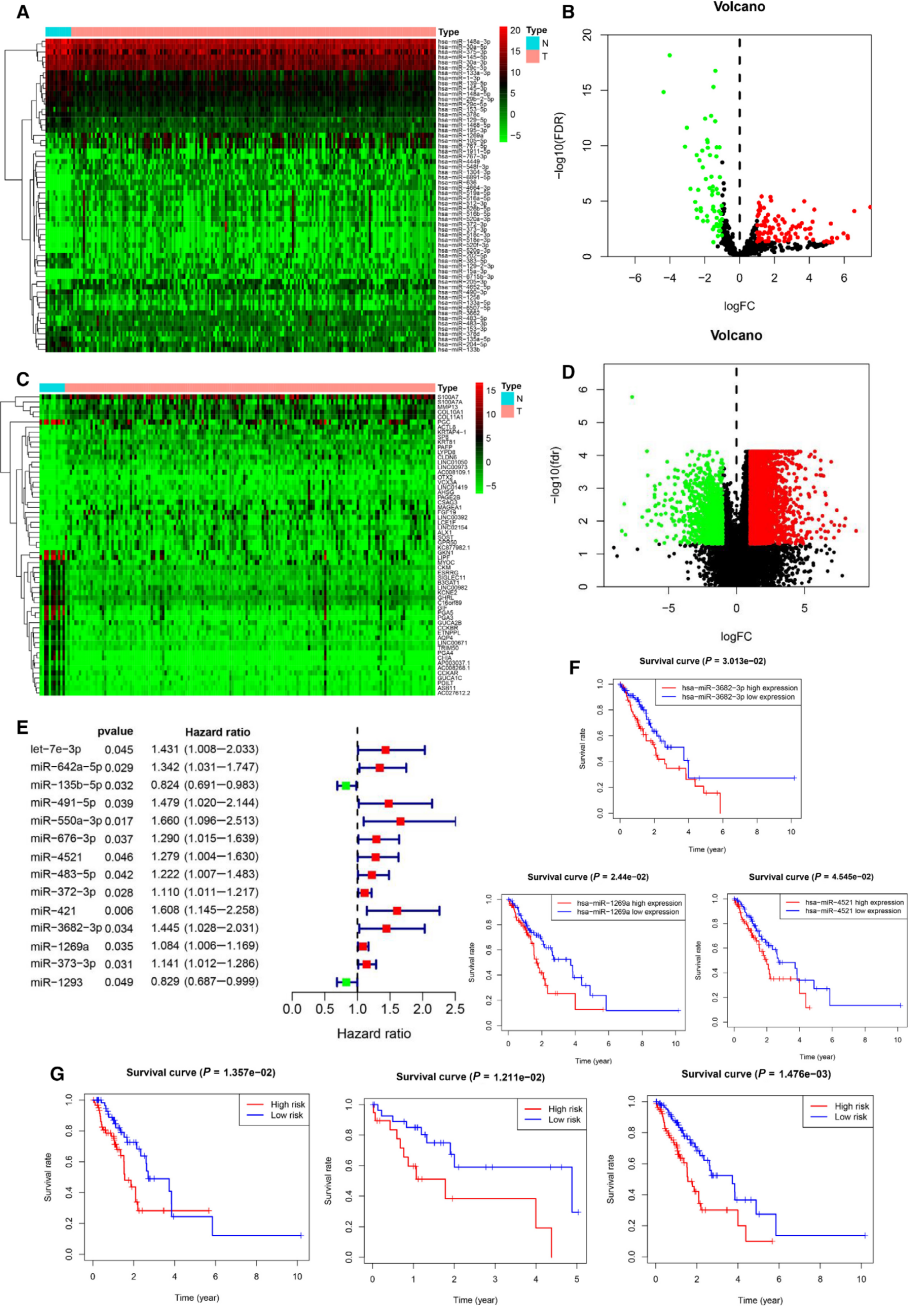
Differentially expressed miRNAs (DEMs) and differentially expressed genes (DEGs)in EC. (A) Top 20 of up‐regulated and down‐regulated DEMs in EC. (B) Volcano plot of DEMs in EC. (C) Top 20 of up‐regulated and down‐regulated DEGs in EC. (D) Volcano plot of DEGs in EC. (E) Forest plot of univariate Cox analysis of DEMs filtered 14 miRNAs. (F) Three miRNAs associated with OS in EC patients using Kaplan–Meier curves and log‐rank tests. (G) Kaplan–Meier curves validated and evaluated three miRNA signature in the training group (left panel), test group (median panel), and all group (right panel).

Here, we explored the DEMs and mRNA (DEGs) based on the TCGA‐ESCA dataset, including 185^tumor^/13^normal^ samples and 160^tumor^/11^normal^ samples, respectively. The clinical information of these patients including age, gender, stage, T stage, lymph node invasion, and metastasis information was also downloaded. As results, the top 30 up‐ and down‐regulated miRNAs are shown in Fig 1A. The 162 differentially expressed miRNAs were identified according to the criteria of FDR < 0.05 and |log_2_FC| > 1.0 (Fig. 1B). Similarly, 4555 differentially expressed mRNAs were detected by the ‘edgeR’ package and shared similar filter criteria with DEMs (Fig. 1C,D).

### 3.2 Construction and evaluation of three miRNA‐based signature for prognostic prediction in ESCC

**Fig. 2:**
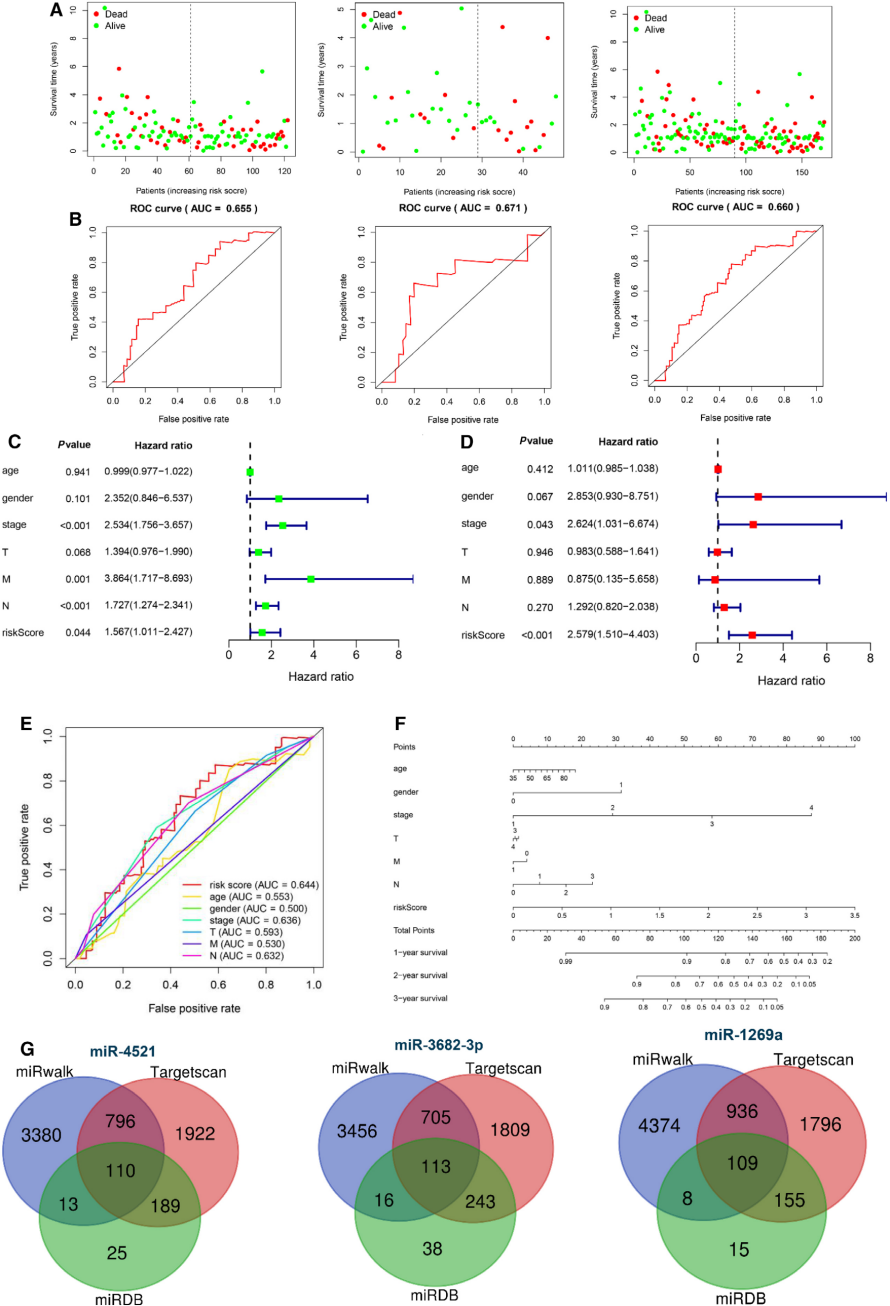
Validation and evaluation of the predictive three miRNA signature and prediction of miRNA target genes. (A) Survival status of high‐ and low‐risk patients in training group (left panel), test group (median panel), and all group (right panel). Red dots represent death and green dots represent alive. (B) The ROC of 3‐year dependent curve in training group (left panel), test group (median panel), and all group (right panel). (C‐D) Forest plot of univariate (C) and multivariate (D) Cox regression analysis showed the risk score was an independent risk factor compared with other clinical features. (E) Comparison of risk score and other clinical characteristics in predicting the accuracy of patient prognosis by ROC curve. (F) Nomogram of three miRNA‐based signature for prediction of patient prognosis in EC. (G) Venn diagram of target genes for three miRNAs in EC.

Patients with mature miRNA expression information were randomly grouped into training and test datasets with a ratio of 0.7. After performing a univariate Cox regression analysis, we identified 14 miRNAs that were associated with poor patient prognosis in the training group (Fig. 1E). Subsequently, multivariate Cox regression analysis followed by LASSO analysis selected three miRNAs (miR‐4521, miR‐3682‐3p, and miR‐1269a) to construct prediction model with their coefficient as follows: miRNA risk score = (0.26 × expression of miR‐4521) + (0.34 × expression of miR‐3682‐3p) + (0.07 × expression of miR‐1269a). The Kaplan–Meier curve pointed out that the high expressions of miR‐4521, miR‐3682‐3p, and miR‐1269a were associated with poor prognosis of EC patients (Fig. 1F). According to the median value grouping of the miRNA risk score, we found a high‐risk group in the training dataset exhibiting an overall poor survival rate compared with the low‐risk group (Fig. 1G, left panel). Meanwhile, similar significant results were found in the test and entire groups (Fig. 1G, median, and right panel). The survival status in three groups showed that the high‐risk score patients had higher mortality rates than the low‐risk group (Fig. 2A). The AUC of the ROC curve for three miRNA signature (Fig. 2B) elucidated better efficiency in EC patient survival risk prediction. Univariate and multivariate Cox regression analyses indicated that three miRNA signature‐based risk score could serve as an independent risk factor for OS of EC patients compared with other clinical features, such as age, gender, T stage, lymph node invasion, and metastasis (Fig. 2C,D). Thus, results indicated a high possibility that this risk model could serve as a prognostic marker in the future. The ROC curve for risk model and nomogram is presented in Fig. 2E,F.

### 3.3 Exploration of target genes and further functional enrichment analysis

**Fig. 3:**
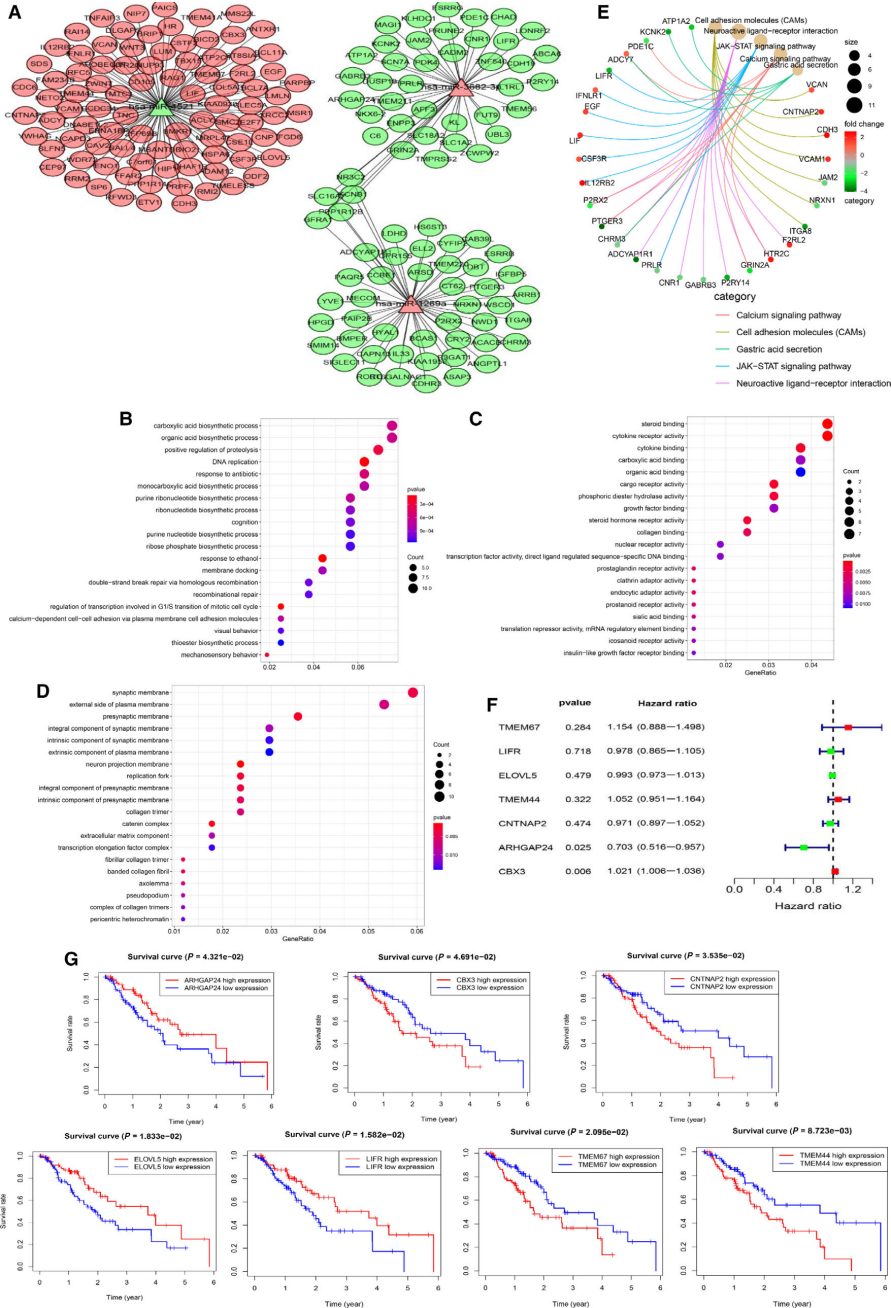
Functional analysis for three miRNA‐based signature and its target genes. (A) Network showing mRNAs negatively regulated by miRNAs. Red denotes up‐regulated; green denotes down‐regulated. (B‐D) Bubble plots showing GO analysis of target genes for BP (B), CC (C), and MF (D). (E) KEGG pathway analysis of the pathway‐gene network presented by cnetplot. (F) Univariate Cox analysis of seven target genes in EC. (G) The association of seven target genes with patients’ prognosis of EC.

To further explore the target genes for the three miRNAs, we applied three prediction databases including miRDB, TargetScan, and miRWalk to enhance the reliability of bioinformatics analysis. The overlapping target genes in the Venn diagram implicated 110, 113, and 119 genes presented in three databases as potential target genes for miR‐4521, miR‐3682‐3p, and miR‐1269a, respectively (Fig. 2G). Further filtration of target genes using the negative correlation criteria [(a) miRNA should be targeted to the genes; (b) the gene expression should be opposite to miRNA expression; (c) the target genes should belong to DEGs)] was presented between the expression level of miRNAs and corresponding target genes. As a result, 177 genes were identified including 91 up‐regulated genes and 86 down‐regulated genes. The subnetworks of the regulation relationship between three miRNAs and their target genes are shown in Fig. 3A. Taken together, these results provided with promising miRNAs and its target genes for further analysis.

After selecting the target genes, potential mechanisms promoting EC progression were detected. The GO analysis results including BP, cellular component (CC), and molecular function (MF) are shown in Fig. 3B–D. Through BP analysis, the carboxylic acid biosynthetic process, as well as the organic acid biosynthetic process, was the most enriched in EC progression. Some of these BPs have not been reported involved in the progression of EC. The CC analysis contained the synaptic membrane and the external side of the plasma membrane. Moreover, the MF analysis mainly contained steroid binding and cytokine receptor activity. Besides, the KEGG analysis demonstrated that cell adhesion molecules (CAMs), neuroactive ligand–receptor interaction, JAK‐STAT signaling pathway, calcium signaling pathway, and gastric acid secretion were the top five enriched pathways (Fig. 3E). Collectively, we identified 177 miRNA‐related target genes and adopted GO and KEGG analysis to elucidated their potential mechanism and provide a novel direction in the treatment and diagnosis of EC.

### 3.4 Seven miRNA signature targets were associated with patient prognosis in ESCC

In total, 177 genes were analyzed by K‐M survival analysis whereby the expression of seven genes [Rho GTPase‐activating protein 24 (ARHGAP24), Chromobox 3 (CBX3), Contactin‐associated protein 2 (CNTNAP2), ELOVL fatty acid elongase 5 (ELOVL5), LIF receptor subunit alpha (LIFR), transmembrane protein 44 (TMEM44), and transmembrane protein 67 (TMEM67)] was revealed to be significantly associated with patient poor prognosis (Fig. 3G). Notably, CBX3, CNTNAP2, TMEM44, and TMEM67 were positively correlated with survival prognosis in EC patients, whereas the high expression level of ARHGAP24, ELOVL5, and LIFR showed poor patient survival (Fig. 3F,G). The role of these genes in progression of EC has not well elucidated except for CBX3. The deep investigation of these genes is therefore urgent which enhance better understanding of EC development and precise treatment. Chromobox 3 (CBX3) was reported to be targeted by miR‐30b and promoted proliferation, migration and inhibit apoptosis of EC cell via activating the JAK2/STAT3 signaling pathway [32]. In summary, we eventually selected seven target genes to carry out a further analysis which both meet a negative correlation with miRNA expression and played an important role in EC patient prognosis.

### 3.5 Association of miRNA signature target genes with immune response, tumor microenvironment, and immune infiltration in EC

**Fig. 4:**
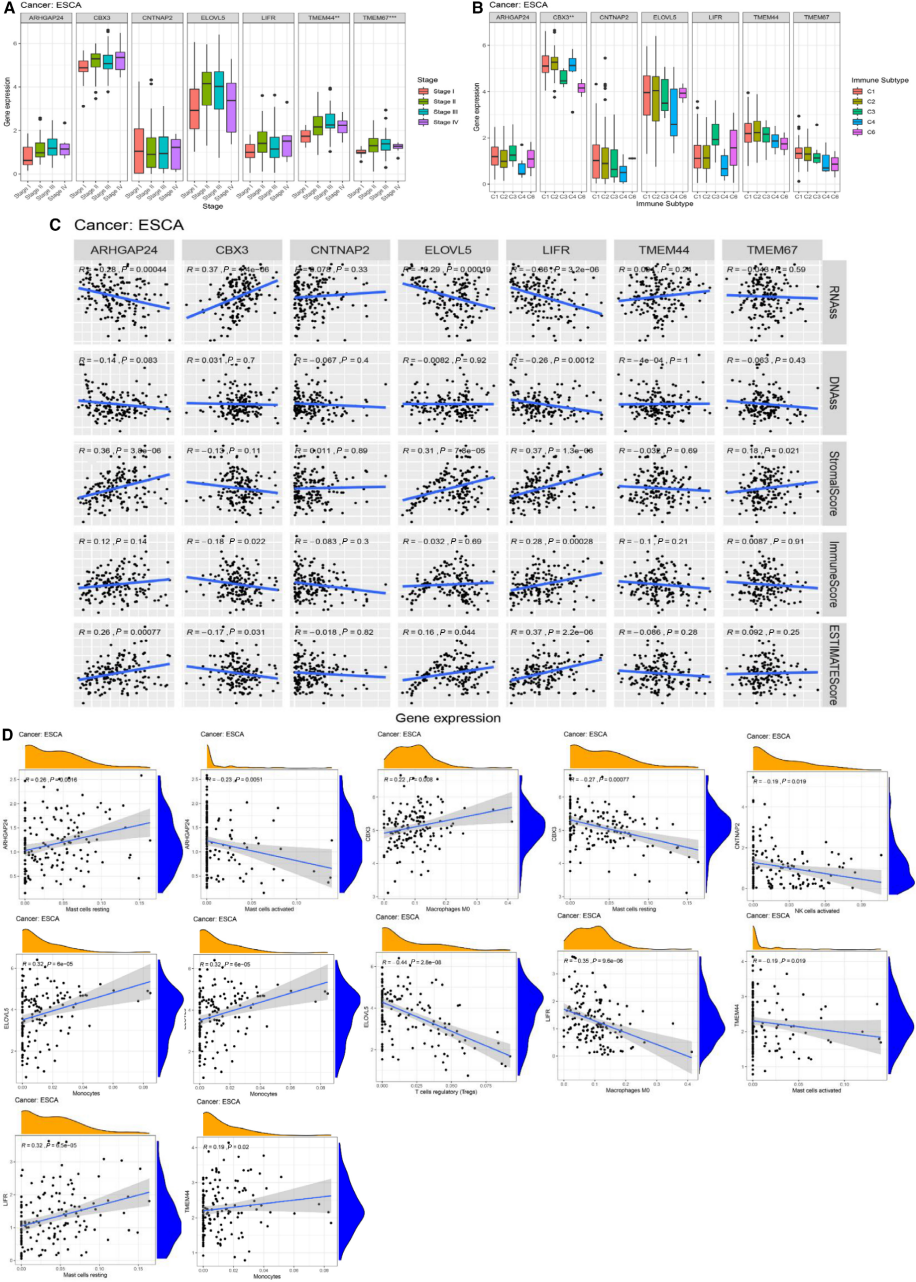
Association of expression of target genes with multiple factors. (A) Association of target genes with clinical stage of EC. (B) Association of target genes expression with immune subtypes in EC patients. (C) Correlation of expression of target genes with cancer stem cell‐like properties (RNAss, DNAss) and TME (Stromal score, Immune score, and ESTIMATE score). *, **, *** represent *P* < 0.05, *P* < 0.01, and *P* < 0.0001, respectively. Spearman correlation analysis was used to calculate the association. (D) The top two significant immune‐infiltrated cells from each target genes are shown. No immune cell infiltration was associated with TMEM67 expression.

Herein, we explored the immunological role of seven miRNA target genes involved in the regulation of immune response and TME in EC. The association between the tumor stage of EC patients and seven target genes is shown in Fig. 4A. The TMEM44 and TMEM67 were found to be significantly correlated with the tumor stage in EC. To understand the association of these target genes with immune components, we examined the correlation of immune infiltration types with target genes in EC. We found six types of immune infiltration in human cancers, including C1 (wound healing), C2 (INF‐r dominant), C3 (inflammatory), C4 (lymphocyte depleted), C5 (immunologically quiet), and C6 (TGFβ dominant). Based on the results in Fig. 4B, high expression of CBX3 was suggested to correlate with C1, C2, and C4 infiltration types, indicating its tumor facilitator role.

The TME plays a crucial role in cancer development and progression. We assessed the association of target genes with the presence of infiltration stromal cells and immune cells through ESTIMATE analysis. Notably, the LIFR showed highest association with stromal score in EC (*r* = 0.37, *P* < 0.001), followed by ARHGAP24 (*r* = 0.36, *P* < 0.001), ELOVL5 (*r* = 0.31, *P* < 0.001), and TMEM67 (*r* = 0.18, *P* = 0.021) (Fig. 4C). In addition, LIFR exhibited highest correlation with immune score in EC (*r* = 0.28 and *P* < 0.001) followed by CBX3 (*r *= −0.18, *P* = 0.022). The ESTIMATE score, combination of stromal and immune score, indicated that high expression levels of LIFR, ARHGAP24, and ELOVL5 were significantly associated with lower tumor purity, whereas CBX3 exerted an inverse effect (Fig. 4C). Thereafter, we applied the CIBERSORT algorithm to reveal the association between the expression of target genes and the infiltration of 22 immune cells. Since the expression of LIFR was strongly associated with TME, we focused on LIFR and found that naive B cells, activated dendritic cells, M0 and M1 macrophages, activated and rest mast cells, and activated memory CD4‐T cells were significantly related with the expression level of LIFR. The top two immune‐infiltrated cell types among seven genes in EC are shown in Fig. 4D and Table 1. Overall, these findings elucidated the impact of target genes on the immune response in the TME, thus could provide an in‐depth understanding of the role of an individual gene in immune therapy.

**Table 1:** The association of immune infiltration cells with target gene expression.

	Correlation (*R*)	*P*‐value
ARHGAP24		
Mast cells activated	0.25	0.0016
Mast cells resting	0.26	0.0016
Monocytes	0.23	0.0053
Neutrophils	0.21	0.0095
CBX3		
B‐cell memory	−0.17	0.038
Macrophages M0	0.22	0.008
Mast cells activated	0.18	0.029
Mast cells resting	−0.27	0.00077
T‐cell CD8	−0.16	0.047
T‐cell follicular helper	0.21	0.012
CNTNAP2		
NK cells activated	−0.19	0.019
ELOVL5		
B‐cell naive	−0.3	0.00021
Dendritic cells activated	0.26	0.0013
Dendritic cells resting	0.28	0.00057
Monocytes	0.32	0.00006
Neutrophils	−0.17	0.037
T‐cell regulatory (Tregs)	−0.44	<<0.0001
LIFR		
B‐cell naive	0.22	0.0079
Dendritic cells activated	−0.27	0.00095
Macrophages M0	−0.35	<<0.0001
Macrophages M1	0.19	0.022
Mast cells activated	−0.26	0.0016
Mast cells resting	0.32	<0.0001
T‐cell CD4 memory activated	0.18	0.024
TMEM44		
Mast cells activated	−0.19	0.019
Monocytes	0.19	0.02
TMEM67	Non	Non

### 3.6 Association of miRNA signature target genes with stem‐like properties and tumor mutation burden in EC

**Fig. 5:**
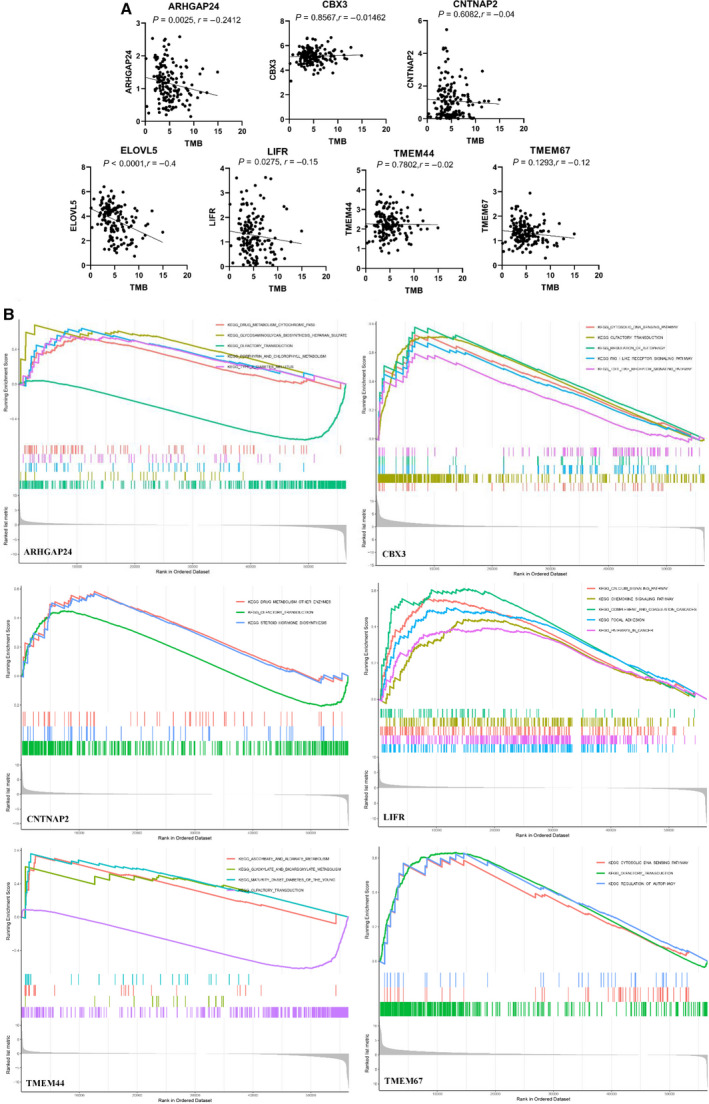
Association of expression of target genes with TMB and its function in tumor progression. (A) The correlation of expression of target genes with TMB in EC patients. (B) GSEA analysis indicating the most enriched pathways of each gene in EC. Spearman correlation analysis was used to calculate the association.

Recently, CSCs have been found to potentially interact with immune cells in the TME and can facilitate tumor progression in many cancers. Our previous study also found that LEF1 of the Wnt signaling pathway could directly bind to the promoter of ID1 and promoted cancer stem‐like properties in ESCC [12]. Consequently, after detecting the role of target genes in TME and immune infiltration, we further explored their role in regulating esophageal CSCs by measuring mRNA expression (RNAss) and DNA methylation pattern (DNAss). As shown in Fig. 4C (upper panel), LIFR was revealed as the only key gene that was significantly associated with both RNAss and DNAss, with *r* = −0.66 and *r* = −0.26, respectively. In addition, ARHGAP24 (*r* = −0.28, *P* < 0.001), CBX3 (*r* = 0.37, *P* < 0.001), and ELOVL5 (*r* = −0.29, *P* < 0.001) were closely related to esophageal CSCs. These results indicated that higher expression of LIFR and ARHGAP24 correlated with reduced cancer cell stemness, which concurred with the fact that increased expression of these genes favored better survival in EC. Previous investigations discovered that the TMB was tightly associated with immunotherapy in many cancer types (16, 17). For instance, Yuan et al. reported that TMB was correlated to Tregs cell infiltration and served as an independent risk factor for EC patient prognosis, suggesting TMB as a prognostic marker for EC patients. As shown in Fig. 5A, ELOVL5 (*r* = −0.398, *P* < 0.001) demonstrated the highest correlation with TMB in EC patients, followed by ARHGAP24 (*r* = −0.26, *P* < 0.001) and LIFR (*r* = −0.17, *P* = 0.035). In summary, these results indicated that lower expression levels of ELOVL5, ARHGAP24, and LIFR promoted a higher TMB, suggesting the three genes as potential biomarkers for immune therapy. Meanwhile, we detected multiple functions of individual target genes in EC to identify their potential mechanisms in the regulation of immune response and stem‐like properties. Following GSEA analysis, the LIFR were highly enriched in the calcium signaling pathway, chemokine signaling pathway, complement and coagulation cascades, focal adhesion, and pathway in cancer (Fig. 5B). The GSEA results of the rest of the genes whose pathway items were more than three are shown in Fig. 5B. With deep insight into these signaling pathways, it might reveal potential mechanisms involved in tumor development and knowledgeable understanding of miRNA‐based signature networks in target treatment.

### 3.7 The immune landscape of target genes in pan‐cancer

**Fig. 6:**
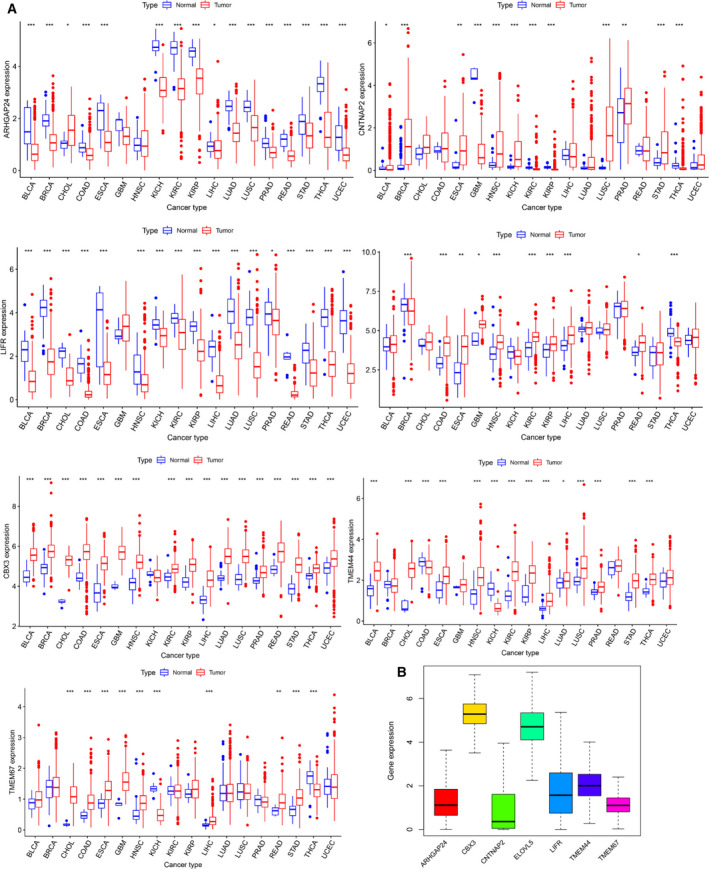
Expression of pan‐cancer target genes. (A) Boxplot showing the expression level of target genes in tumor tissue compared with normal tissue in 18 cancer types which were composed of at least five normal samples. (B) Boxplot showing the expression distribution of target genes across pan‐cancer.

From the aforementioned results, we suggested an important immunological role of target genes in EC. To further understand the intrinsic expression pattern of seven target genes and their association with patients' survival, TME, immune response, stem‐like properties, and TMB in pan‐cancer, we downloaded 33 cancer types from the TCGA dataset for analysis. First, we examined the landscape of the expression pattern of the target genes. The striking inter‐ and intratumor heterogeneity regarding the expression levels of target genes is shown in Fig. 6A. For instance, ARHGAP24 and LIFR showed higher intertumor heterogeneity with some tumors expressed in significantly low levels (COAD, READ), while others were characterized by high expression levels (KIRC, KICH). The other target genes showed degrees of great heterogeneity. Meanwhile, the expression distribution of seven target genes across all 33 cancer types is shown in Fig. 6B. These results showed that intrinsic differences in the expression of target genes exist between different tumors, suggesting the need for in‐depth research on each tumor.

**Fig. 7:**
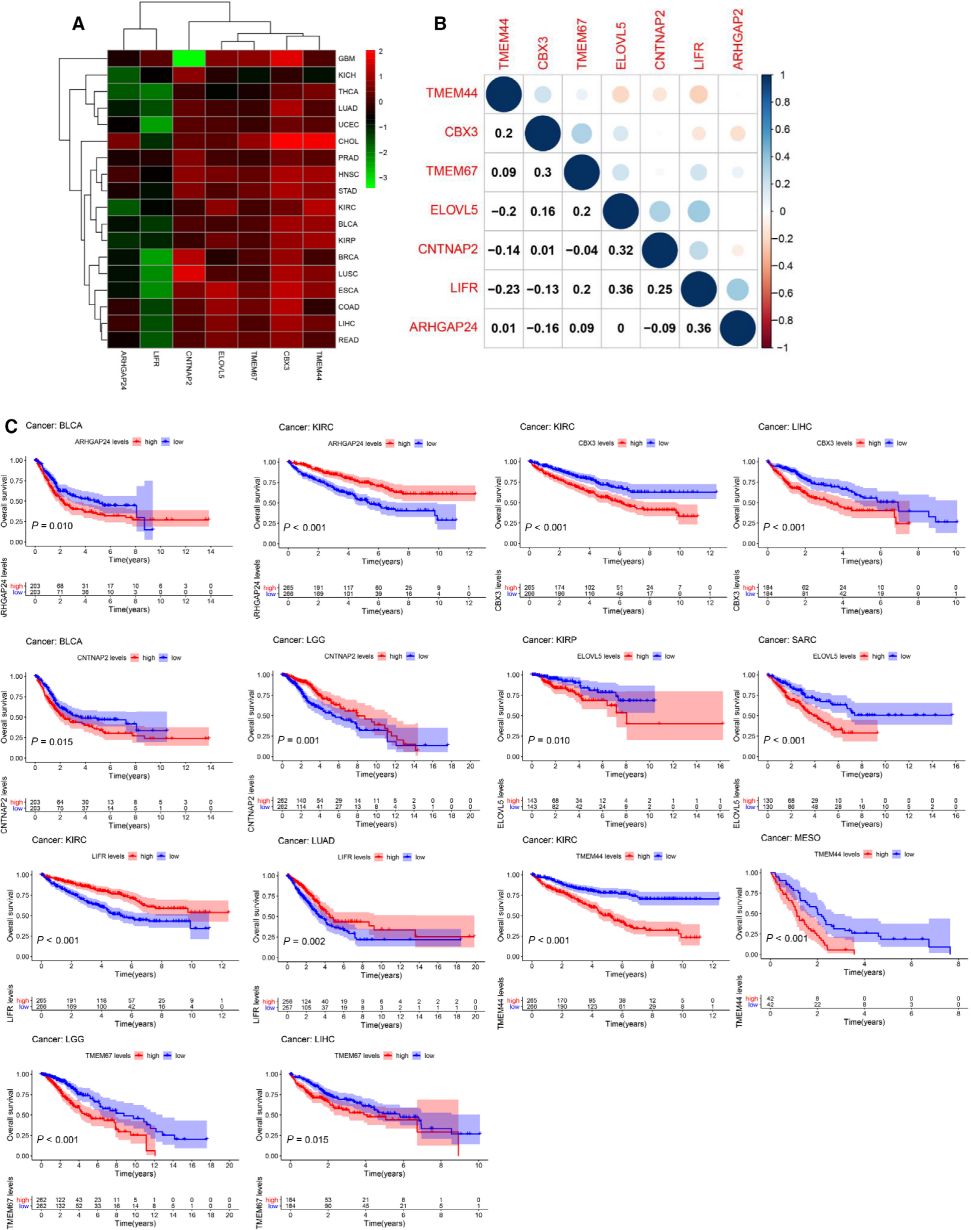
Expression levels of seven target genes in cancers and adjacent normal tissues. (A) Heatmap showing the expression level of target genes in tumor tissue compared with normal tissue in 18 cancer types which were composed of more than five normal samples. (B) Correlation plot based on Spearman correlation analysis showing the correlation of gene expression among the seven target genes across all 33 cancer types. (C) The association of seven target genes with patients’ prognosis in pan‐cancer. The representative top two cancer types among target genes are shown according to the *P*‐value.

Then, we further explored the expression levels of target genes in all 33 tumor types including EC, and selected 18 tumors with the normal sample more than five for further study. The expression levels of TMEM44, TMEM67, CBX3, and ELOVL3 in tumor tissue were higher than in normal tissue, which was consistent with the trend in EC. The direction of the altered expression of LIFR, ARHGAP24, and CNTNAP2 varied in each cancer type. Whereas CNTNAP2 was overexpressed in 18 tumors, the LIFR and ARHGAP24 expression levels were down‐regulated with few exceptions (Fig. 7A). Moreover, we found that the pairs LIFR and ELOVL5 (*r* = 0.36, *P* < 0.01), ARHGAP24, and LIFR (*r* = 0.36, *P* < 0.01) had the highest association, indicating that they may share common functions (Fig. 7B).

In previous results of this study, we found seven target genes were significantly associated with the OS of EC patients. Herein, we were prompted to further investigate which target genes promoted or inhibited patient survival in which cancer type through K‐M curve and Cox regression analysis across 32 cancer types. The top two cancer types from the seven target genes are shown in Fig. 7C except for EC. Furthermore, we used univariate Cox proportional hazard regression models to determine the altered expressions of seven target genes associated with patient survival, and the direction of association varied depending on cancer types (Fig. 9A).

**Fig. 8:**
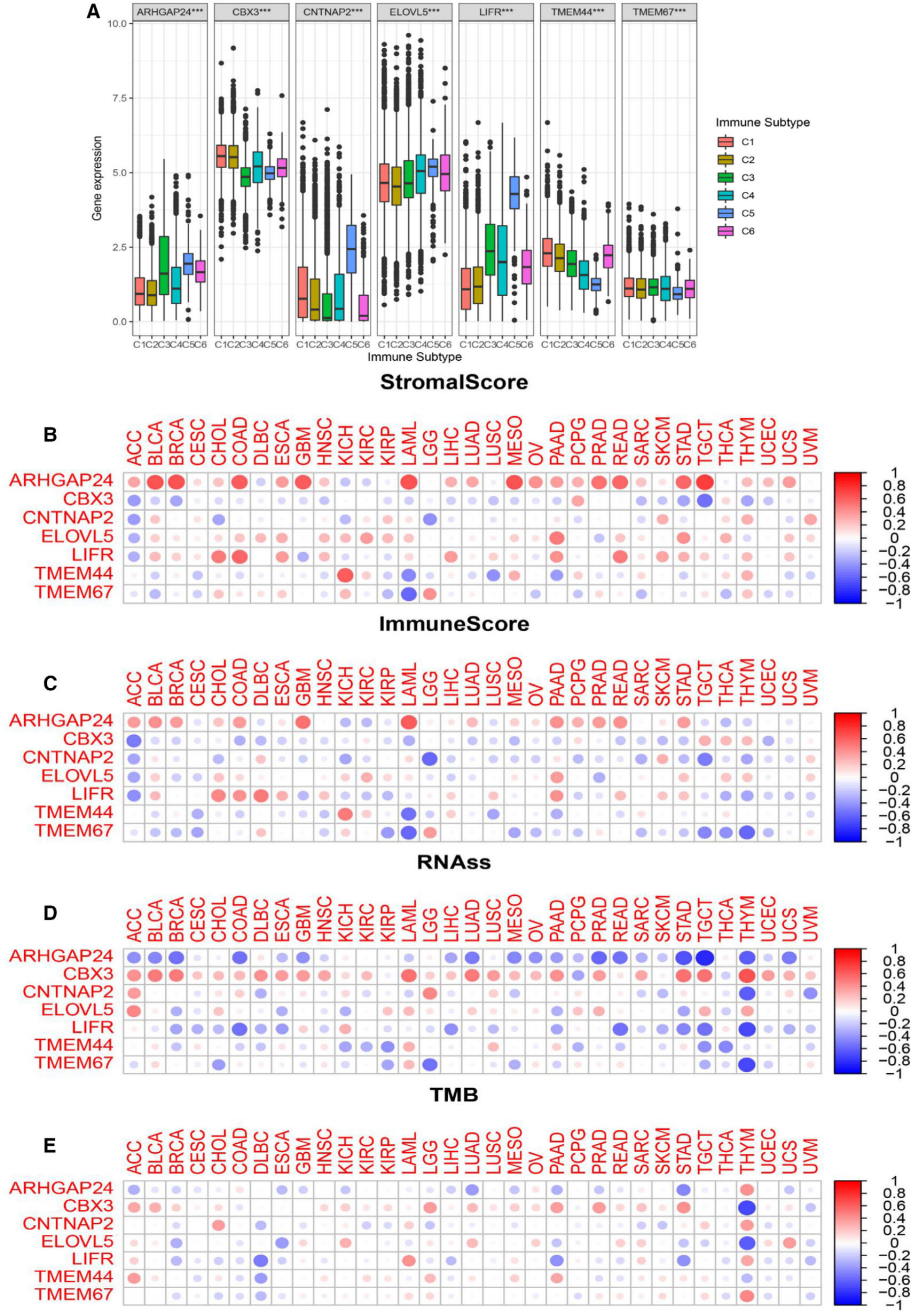
Association of expression of target genes with immune subtypes, TME, cancer stemness, and TMB in pan‐cancer. (A) Association of expression of target gene with immune infiltrate subtypes across all the cancer types tested with ANOVA. (B‐C) Correlation matrix between TME stromal scores (B) and immune scores (C) and target genes expression by ESTIMATE algorithm. (D) Correlation matrix plots showing the association between target genes expression and cancer stemness RNAss score.

**Fig. 9:**
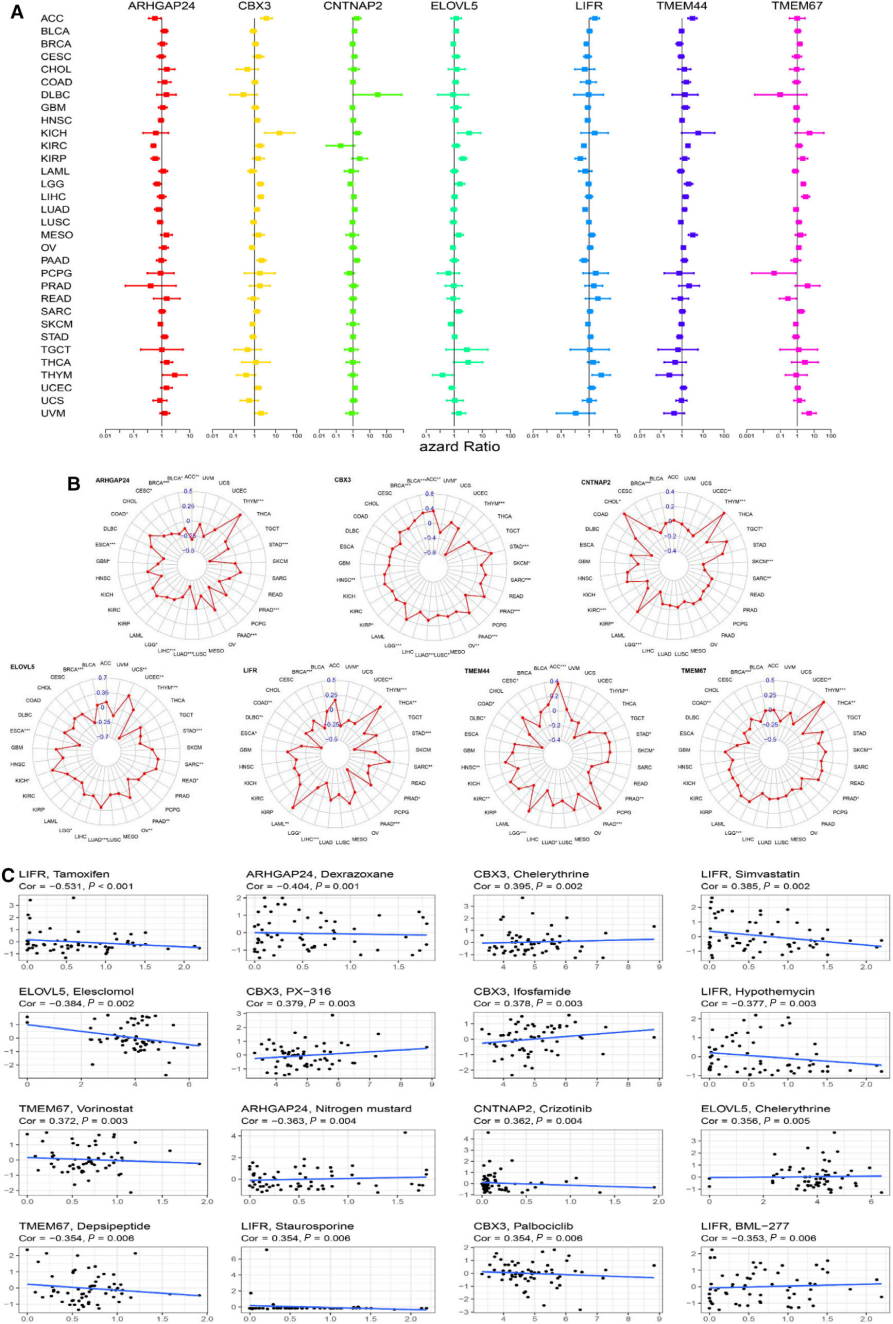
Association of expression of target genes with patient prognosis, drug sensitivity, and TMB in pan‐cancer. (A) The forest plots with 95% CIs and HRs for OS for different cancer types in pan‐cancer. (B) Radar graph indicating the detailed information of the association between target gene expression and TMB in pan‐cancer. (C) The scatter plot indicates the correlation between target gene expression and drug sensitivity (the *z*‐score of the CellMiner interface) for the Pearson correlation test using NCI‐60 cell line data. Top 16 associations are shown, ordered by *P*‐value.

The seven target genes have been proved to be associated with the TME, immune subtype, immune infiltration, and stem‐like properties in EC. However, there was also a lack of relevant researches that focused on the landscape of miRNA‐based target genes that participates in immune response and tumor progression in pan‐cancer. In this section, we first investigated the association of target gene expression with six immune subtypes. Results indicated that all seven genes were strongly associated with immune subtypes in pan‐cancer (Fig. 8A). More specifically, the pan‐cancer distribution of expression levels of target genes in immune types was mainly consistent with that in EC. For instance, patients showed more invasion and aberrant prognosis subtype (C1, C2, C4) as CBX3 was up‐regulated in pan‐cancer. The landscape of correlation of target genes with the immune and stromal score, cancer stem‐like properties, and TMB is displayed in Fig. 8B–E. Distinctly, the target genes showed various levels of association with these characteristics in different cancer types, but the directions of correlation were mainly similar to EC. Specifically, we found a significantly positive correlation of ARHGAP24 expression with the stromal score in pan‐cancer, whereas it was negatively associated with RNAss. All the seven target genes were strongly associated with RNAss and TMB in THYM; this showed the optimal prediction role of these genes. Although TMEM44 and TMEM67 were less relevant to TME, RNAss, and TMB in EC, they were found to be significant in LAML, KICH, THYM, and DLBC. The radar plots of correlation between each target genes and pan‐cancer are shown in Fig. 9B.

We assessed the influence of target genes on drug sensitivity using CellMiner database, which could facilitate better precision treatment. Drug sensitivity was measured by *z*‐score, and the higher the scores implied that cells were more sensitive to the drug treatment (Fig. 9C). Notably, elevated expression of target genes, especially LIFR, CBX3, and ARHGAP24, was associated with drug resistance in different cell lines to several chemotherapy drugs (Fig. 9C). For example, LIFR was associated with cell resistance to the treatment of tamoxifen (treatment for BRCA). Furthermore, we noticed that different genes had similar associations with the same drug. For example, CBX3 and ELOVL5 were both associated with increased sensitivity of cells to chelerythrine (treatment for STAD).

## 4 Discussion

Esophageal cancer is one of the most aggressive malignant tumors with a high possibility of metastasis and the leading cause of cancer‐related deaths globally [33]. Despite advances in precision diagnosis and treatment, the 5‐year OS rate of EC remains low, especially in East Asia [34]. Therefore, there is an urgent need to develop more specific and sensitive prognostic biomarkers for EC patients. Recently, extensive studies found that miRNAs could regulate downstream gene expression, playing an important role in tumor development and prognosis [35]. In particular, recent researches have revealed that miRNA expression profiles could promote EC progression and metastasis [36, 37]. In our study, we found that miR‐34a‐5p directly targeted LEF1 and inactivated the Hippo signaling pathway, which inhibited invasion and migration of ESCC [5]. MiRNAs could widely be used as prognostic biomarkers in various types of cancers, including miR‐191, miR‐1908, and miR‐217. However, due to the insufficient specificity and sensitivity, more miRNA signatures and their mechanisms in promoting cancers need to be explored, especially by developing multiple miRNAs combined signature. Due to the merits of multiple miRNA‐based signature and the lack of relevant studies in EC, we identified a prognostic miRNA signature that enhanced prediction of patient prognosis in EC patients and guides on precision treatment.

In this study, bioinformatics analysis was applied to identify the optimal prognostic miRNA signature. We downloaded mature miRNA and mRNA expression profiles, and corresponding patient clinical information on EC from the TCGA database. Exactly 162 differentially expressed miRNAs (DEMs) and 4555 differentially expressed genes (DEGs) were identified using the ‘edgeR’ package. Patients were randomly classified into the training and test groups. A three miRNA‐based prediction signature including miR‐4521, miR‐3682‐3p, and miR‐1269a was obtained in the training group by univariate Cox and multivariate Cox analysis. The miRNA signature model was validated in the test and the entire group. Kaplan–Meier analysis showed that the high‐risk score group exhibited poorer patient prognosis of EC compared with the low‐risk group. The efficacy of this prediction model was evaluated by ROC curve and the AUC showed a better prediction power. Moreover, we found that a risk score based on the miRNA signature model was an independent risk factor compared with other clinical features, such as the T stage, lymph node invasion, and metastasis. Among these three miRNAs, studies have reported on the role of three signature miRNAs in the development and progression of various cancers, especially for EC. This indicated that in‐depth insight into three miRNAs is recommended in the future. A study by Feng and colleagues demonstrated that the miR‐4521‐FAM129A axis played an important role in the progression of renal cell carcinoma [38]. However, MiR‐1269a could directly bind to the downstream target of SOX6 and act as an onco‐miRNA in the progression of non‐small‐cell lung cancer [39].

To further understand the regulatory mechanisms involved in the three miRNA signature, we explored the target genes of three miRNAs by an intersection with three different databases (miRDB, TargetScan, and miRWalk). Then, we intersected the prediction genes with differentially expressed genes from TCGA. The intersection target genes which were negatively correlated with the expression of these miRNAs were finally selected. Furthermore, the GO and KEGG analyses were applied after 177 target genes were obtained. Results indicated that the carboxylic acid biosynthetic process and organic acid biosynthetic process were the most enriched process involved in EC progression. Chen *et al*. found that citric acid as an important organic acid prevent EC cell growth via inhibiting cell proliferation and apoptosis, which could serve as a novel therapeutic target for the treatment of EC [40]. The CC analysis was mainly contained synaptic membrane and external side of the plasma membrane. MF analysis mainly contained steroid binding and cytokine receptor activity. The KEGG analysis showed that CAMs, neuroactive ligand–receptor interaction, JAK‐STAT signaling pathway, calcium signaling pathway, and gastric acid secretion were the top five enriched pathways. Elsewhere, You et al. demonstrated that pJAK‐1 and pSTAT‐3 were up‐regulated in esophageal squamous cell carcinoma patients and associated with aberrant clinicopathological features, which promoted progression and invasion of ESCC [41]. It was also reported that the calcium signaling pathway not only played a crucial role in progression and sensitivity to cell death but also in the establishment and maintenance of multidrug resistance and the TME [42]. These signaling pathways critically impact on various tumors in varying degrees, which might explain potential mechanisms that lay behind our three miRNA‐based model.

A recent study reported that cancer patients could be grouped into six immune infiltration subtypes [43], including C1 (wound healing), C2 (INF‐r dominant), C3 (inflammatory), C4 (lymphocyte depleted), C5 (immunologically quiet), and C6 (TGFβ dominant). The immune subtypes showed a strong association with OS and progression‐free survival, with C3 owing the optimal survival and C1 and C2 representing poor prognosis. Meanwhile, six types of immune subtype were associated with several somatic mutations, such as copy number variation and homologous recombination deficiency. In our study, we explored the association of seven target genes with immune infiltration subtypes according to patient immune types in the TCGA‐ESCA dataset. High expression of CBX3 was found to be strongly associated with a more aggressive subtype of C1, C2, and C6, implicating CBX3 as a potential tumor promoter in EC.

A wealth of studies has revealed that TME plays a crucial role in tumor metastasis and progression [44]. Cancer cells and noncancer cells are made up of TME, including stromal cells, immune cells, extracellular matrix, and so on [45]. The stromal cells and immune cells in TME potentially affect immune therapy response. Previous studies showed that stromal cells are related to the exocrine phenotype of T cells in bladder cancer and the existence of immune cells facilitated the elimination of tumor cells via various mechanisms [46]. Other reports revealed that immune activity and immune cell infiltration in TME could be quantified by tumor gene expression profiles [47]. Therefore, digging into tumor gene expression profiles could explore the relationship between TME and patient prognosis as well as to evaluate the immune treatment response. In the present study, we found that LIFR showed the highest association with stromal score in EC (*r* = 0.37, *P* < 0.001), followed by ARHGAP24 (*r* = 0.36, *P* < 0.001), ELOVL5 (*r* = 0.31, *P* < 0.001), and TMEM67 (*r* = 0.18, *P* = 0.021). In addition, LIFR exhibited the highest correlation with immune score in EC with *r* = 0.28 and *P* < 0.001, followed by CBX3 (*r *= −0.18, *P* = 0.022). ESTIMATE score demonstrated that high expression levels of LIFR, ARHGAP24, and ELOVL5 were significantly associated with lower tumor purity, whereas CBX3 exerted an inverse effect. Moreover, LIFR was significantly associated with naïve B cells, activated dendritic cells, M0 and M1 macrophages, activated and rest mast cells, and activated memory CD4‐T cells infiltration. Taken together, these findings showed that low expression of LIFR signified higher immune cells and stromal cell infiltration in TME and lower tumor purity in EC. More specifically, as LIFR is down‐regulated by miR‐3682‐3p and less M1 macrophages infiltrate into TME, the ability of EC progression was enhanced, suggesting the role of LIFR in tumor progression. Similar results were found with ARHGAP24 and ELOVL5.

Cancer stem cells were generated during cancer progression whereby tumor cells were transformed from differentiated phenotype to progenitor and consequently acquired stem cell‐like features [48]. We also reported that CSCs were associated with immune response and TME. For instance, TAMs could enhance the proliferation of CSCs derived from hepatocellular carcinoma through IL‐6‐induced STAT3 activation. STAT3 further stimulated the production of cytokines, forming a positive feedback loop that promoted cancer stem cell self‐renewal [49]. Moreover, CSCs could drive tumorigenesis and progression by regulating the activity of immune cells. Glioblastoma CSCs have been shown to express various cytokines (including colony‐stimulating factor, TGF‐β, and macrophage inhibitory cytokines), thus promoting polarization of macrophages to M2 type [38]. Therefore, an in‐depth understanding of the role of target genes in the crosstalk between CSCs, immune response, and TME is a promising aspect for cancer treatment. In our study, LIFR was found to be significantly associated with both RNAss and DNAss, with *r* = −0.36 and *r* = −0.26, respectively. ARHGAP24 (*r* = −0.28, *P* < 0.001), CBX3 (*r* = 0.37, *P* < 0.001), and ELOVL5 (*r* = −0.29, *P* < 0.001) were also closely related to esophageal CSCs.

Due to many limitations of standard therapy for EC treatment, promising new roads for EC treatment have been surged recently, including immune checkpoint inhibitors, tumor vaccine, and adoptive T‐cell treatment [50]. However, the EC immunotherapies also result in mixed outcomes, mainly caused by the absence of an efficient biomarker to predict immune response [51]. TMB, which was related to the generation of neoantigens, has been applied to predict the response to PD‐1 and PD‐L1 blockages in various tumors. Herein, we demonstrated that ELOVL5 (*r* = −0.398, *P* < 0.001) showed the highest correlation with TMB in EC patients, followed by ARHGAP24 (*r* = −0.26, *P* < 0.001) and LIFR (*r* = −0.17, *P* = 0.035). Of note, these observations demonstrated that lower expression levels of ELOVL5, ARHGAP24, and LIFR promoted higher TMB, an implication that these three genes and the relevant miRNAs are potential biomarkers for immune therapy.

In summary, we elucidated the immunological role of seven target genes and found that LIFR, ARHGAP24, ELOVL5, and CBX3 were extremely prominent in EC. More specifically, taking LIFR as an example, we revealed that miR‐3682‐3p was up‐regulated in EC and associated with poor patient prognosis. Additionally, high expression of miR‐3682‐3p contributed to low LIFR expression and was correlated with various immune cell infiltration and stromal components in the TME as well as enhancing stem‐like properties in EC. Low expression of LIFR was also correlated with high TMB. Therefore, targeting the miR‐3682‐3p‐LIFR axis or other target gene axis might impede tumor development in EC. Taken together, these findings showed that the miRNA/mRNA‐based signature could serve as important biomarkers for tumor progression and prediction of the immune response in EC.

Although the role of miRNA/mRNA signature is comprehensively elucidated in EC, no systemic study has been conducted on these genes in different human cancers. Each gene was only studied in a few types of cancers and most of those studies used cell lines and animal models. Next, we wound probe into the immunological role of miRNA signature in the pan‐cancer analysis. We downloaded 32 cancer expression profiles, mutation, and clinical information from the TCGA dataset except for ESCA. Firstly, the landscape of the expression pattern of target genes was assessed, and results showed the striking inter‐ and intratumor heterogeneity regarding the expression levels of target genes. However, the directions of the altered expression of these genes in pan‐cancer were mainly consistent with that of EC, which indicated a similar function of these genes in pan‐cancer. We further examined the association between the expression of the target genes with patient OS in 33 cancer types and found that the direction of association is cancer type‐dependent. Moreover, LIFR was mainly negatively associated with the prognosis of ACC, KIRC, KIRP, LAML, LUAD, while the remaining genes had an antagonistic association with survival (both an advantage and a disadvantage). Interestingly, the results demonstrated that all target genes were significantly associated with immune infiltrating subtypes in the TME, notably, CBX3 and TMEM44 correlated with more aggressive subtypes (C1, C2, C6), whereas LIFR, ARHGAP24, and ELVLO5 associated with more protective subtypes (C3, C5). Target genes were also correlated with the level of infiltration of immune cells and stromal cells, cancer stemness, and TMB at various degrees based on ESTIMATE and other algorithms. For instance, the association between the expression of target genes and tumor stemness score as well as with the drug sensitivity score indicated that CBX3 may mainly play tumor promotor roles during tumorigenesis as they are positively associated with tumor stemness and drug resistance scores. These findings mainly coincided with the observations reported in EC, suggesting that these genes may be utilized as direct therapeutic targets or help in predicting the efficacy of immune checkpoint modulators in cancer patients.

## 5 Conclusion

This study demonstrated that the three miRNA‐based signature and target genes were primarily associated with patient prognosis and played important immunological roles in cancer progression and metastasis, especially for EC. Therefore, we presented potential therapeutic targets for precision cancer treatment.

## Conflicts of interest

The authors declare no conflict of interest.

## Author contributions

YZ performed the experiments, conducted the statistical analysis, conceived and designed the study, analyzed the data, and wrote the manuscript. XYW and LX analyzed the data and made final approval of manuscript. LX and SN conducted the statistical analysis and wrote the manuscript. HZC and CGL conceived and designed the study, and made financial support. All authors read and approved the final manuscript.
